# The effects of knee arthroplasty on walking speed: A meta-analysis

**DOI:** 10.1186/1471-2474-13-66

**Published:** 2012-05-06

**Authors:** Hamid Abbasi-Bafghi, Hamid R Fallah-Yakhdani, Onno G Meijer, Henrica CW de Vet, Sjoerd M Bruijn, Li-Yong Yang, Dirk L Knol, Barend J Van Royen, Jaap H van Dieën

**Affiliations:** 1 Research Institute MOVE, Faculty of Human Movement Sciences, VU University, Amsterdam, The Netherlands; 2 Department of Physical Education and Sports Science, Yazd University, Yazd, Iran; 3 Orthopedic Biomechanics Laboratory, Second Affiliated Hospital of Fujian Medical University, Quanzhou, Fujian, P.R. China; 4 Department of Rehabilitation, Fujian Medical University, Fuzhou, Fujian, P.R. China; 5 EMGO Institute for Health and Care Research, VU University medical center, Amsterdam, The Netherlands; 6 Department of Epidemiology and Biostatistics, VU University medical center, Amsterdam, The Netherlands; 7 Motor Control Laboratory, Research Center for Movement Control and Neuroplasticity, Department of Biomedical Kinesiology, KU, Leuven, Belgium; 8 Department of Orthopedics, First Affiliated Hospital of Fujian Medical University, Fuzhou, Fujian, P.R. China; 9 Union Hospital Affiliated to Fujian Medical University, Fuzhou, Fujian, P.R. China; 10 Research Institute MOVE, VU University medical center, Amsterdam, The Netherlands

**Keywords:** Knee osteoarthritis, Knee arthroplasty, Walking speed, Meta-analysis, Meta-regression analysis

## Abstract

**Background:**

Patients with knee osteoarthritis patients have problems with walking, and tend to walk slower. An important aim of knee arthroplasty is functional recovery, which should include a post-operative increase in walking speed. Still, there are several problems with measuring walking speed in groups of knee osteoarthritis patients. Nevertheless, test-retest reliability of walking speed measurements is high, and when the same investigators monitor the same subjects, it should be possible to assess the walking speed effects of knee arthroplasty. The present study reports a meta-analysis of these effects.

**Methods:**

A total of 16 independent pre-post arthroplasty comparisons of walking speed were identified through MEDLINE, Web of Science, and PEDro, in 12 papers, involving 419 patients.

**Results:**

For 0.5–5 months post-operatively, heterogeneity was too large to obtain a valid estimate of the overall effect-size. For 6–12 and 13–60 months post-operatively, heterogeneity was absent, low, or moderate (depending on estimated pre-post correlations). During these periods, subjects walked on average 0.8 standard-deviations faster than pre-operatively, which is a large effect. Meta-regression analysis revealed significant effects of time and time squared, suggesting initial improvement followed by decline.

**Conclusion:**

This meta-analysis revealed a large effect of arthroplasty on walking speed 6–60 months post-operatively. For the first 0.5–5 months, heterogeneity of effect-sizes precluded a valid estimate of short-term effects. Hence, patients may expect a considerable improvement of their walking speed, which, however, may take several months to occur. Meta-regression analysis suggested a small decline from 13 months post-operatively onwards.

## Background

Patients with knee osteoarthritis have problems walking, and tend to walk slower than controls. Functional recovery is an important aim of unicompartmental (UKA) or total (TKA) knee arthroplasty in patients with symptomatic osteoarthritis, and walking speed may be a useful variable for assessing the functional effects of knee arthroplasty.

Over the last years, walking speed has received considerable attention in the literature. In elderly subjects, a decrease in comfortable walking speed may be a sign of co-morbidity [1], or even impending death [2-5]. In knee osteoarthritis, decreased walking speed is associated with joint space narrowing [6], increased concentrations of inflammation mediators [7], and pain [8]. After arthroplasty, walking speed is expected to increase [9], but in a longitudinal study, pain reduction did not lead to increased walking speed in knee osteoarthritis patients with new co-morbid conditions [10]. Hence, walking speed may not only be used as a simple instrument to monitor post-operative recovery, but also as a screening tool for co-morbidity.

Unfortunately, there are problems in measuring walking speed in groups of knee osteoarthritis patients. Questionnaires are often used, but may be insufficiently valid, since post-operative patients tend to overestimate their own performance when pain has decreased [11,12]. Clearly, walking speed needs to be assessed objectively. However, the methodology of walking tests has a major impact on results. Analyzing twin pairs, Pajala et al. [13] concluded that about half the variance of measured walking speed derived from the environment and the methodology of walking tests. In a review of clinical studies, Graham et al. [14] confirmed the latter point, and argued that “subtle differences in … instructions” (p. 870) may affect the results. In other words, even factors the researchers are hardly aware of, such as the timbre of a voice or clutter in the lab, may co-determine self-selected walking speed. Finally, there is the problem of the notion of patient “groups”. As to the primary diagnosis, such a group may be homogeneous, but over 80% of knee osteoarthritis patients have one or more co-morbid conditions [15], most of which affect walking speed [16]. Hence, walking speeds in patient groups are almost certainly heterogeneous.

There is a vast amount of literature on prognostic factors in knee arthroplasty. For instance, co-morbidity [17] and higher age [18] may slow down functional recovery, while UKA, in comparison with TKA [19], or the use of a clinical pathway [20], may speed up recovery. In response to all this heterogeneity, Ornetti and co-workers [9] expressed the belief that a valid meta-analysis of walking speed recovery after knee arthroplasty is presently unobtainable. Still, Ornetti et al. reported a mean increase in walking speed of 0.16 m/s (= 0.58 km/h), which is large enough to be clinically meaningful [10], and may well turn out to be statistically significant in meta-analysis.

Test-retest reliability of walking speed is high, with most reported IntraClass Correlations (ICCs) at or above 0.9 [21,22]. Thus, when the same researcher measures the same subjects repeatedly, using the same methodology, and within a reasonably short time interval, values will be similar. Still, in meta-analyses of the walking speed effects of arthroplasty, large between-study variance has to be expected. Meta-regression analysis was developed to deal with this problem, by pinpointing variables that contribute to this variance.

The present study is a meta-analysis, including a meta-regression analysis, of the effects of knee arthroplasty on walking speed. We hypothesized a) large variance in the first period after arthroplasty (due to variability in post-operative recovery), but b) still a clear effect, which, however, c) would decrease after some time (due to co-morbid conditions or an increase in age-related diseases).

## Methods

### Literature search

In August 2009, a search was conducted in MEDLINE, Web of Science, the Cochrane Library, and PEDro, with combinations of the search terms: knee, osteoarthritis, walking, gait, velocity, speed, replacement, arthroplasty, and surgery. Full English reports were included of studies on knee osteoarthritis patients who underwent knee arthroplasty, with mean values and standard-deviations of pre- and post-operative walking speed. To decide on relevance, two members of our research group (H.A.-B. and H.R.F.-Y.) inspected titles and abstracts of all papers, and selected “eligible” studies [23], with reviewer agreement expressed as Cohen’s kappa, and open discussion to resolve disagreement. Two authors (H.A.-B. and O.G.M.) read all eligible papers, established which papers contained the information needed, and made the definitive selection of studies included.

### Data description

Two authors (H.A.-B. and O.G.M.) extracted all relevant data from the papers selected. The number of subjects was registered, plus potentially relevant variables—UKA vs. TKA, distance walked, gender (% male), age, BMI, and any measures of disease severity, co-morbidity, pain, or function. For all studies, self-selected (comfortable) walking speed (mean ± SD) before and after arthroplasty was entered. For descriptive purposes, absolute numbers were used. For walking speed, weighted means were calculated, with standard error of the mean as weighting factor. Since standard-deviations were not always given for age and BMI, these variables were weighted with the number of subjects per study.

### Meta-analyses

Effect-sizes (*ES*s) were calculated as [24]:

(1) $$ES=\frac{M_{\text{post}}-M_{\text{pre}}}{SD_{\text{pre}}}$$

where *M*_pre_ is the mean pre-operative value, *M*_post_ the mean post-operative value, and *SD*_pre_ the pre-operative standard-deviation. The variance of the single-study *ES* is a function of the number of subjects, the actual *ES*, and the within-study correlation, *r*_*P*_, between pre- and post-values [25]. Unfortunately, papers rarely report this correlation, but it can be calculated from quantitative results of repeated measures tests. In the present study, *r*_*P*_ was determined where possible. To establish if lower or higher values of *r*_*P*_ would affect the conclusions, all procedures were subsequently rerun for correlations of 0.0 through 0.9.

The overall *ES* was calculated using standard methods [26], and if the *Q*-statistic for heterogeneity was not significant, a fixed, otherwise a random effects model was applied. The significance of the *ES* was determined with a standard-normal *Z*-test [26]. The clinical literature suggests an initial improvement of function after arthroplasty [27], followed by a plateau [28], and after some time often [28], but not always [29], a decline. This pattern suggests that the short-term, mid-term, and long-term walking speed effects of knee arthroplasty should be differentiated. We plotted all effect-sizes over time, and used this plot to select cutting points between “short-term”, “mid-term”, and “long-term”.

### Meta-regression

To quantitatively determine heterogeneity, *I*^2^ was calculated, i.e., the percentage between-study variance in the total variance of *ES*[30]. The literature suggests 25% as “low” heterogeneity, 50% as “moderate”, and 75% as “high” [31]. Accordingly, if *I*^2^ ≥ 75%, the overall *ES* was regarded as uninterpretable.

Meta-regression was used to assess the impact of relevant factors on *ES* with high heterogeneity [32]. These factors had to be mentioned in at least 10 studies. A random-effects regression model for meta-analysis [33] was implemented in MATLAB 7.0.4 (and a subset of the calculations validated with SAS 9.1, which provided the same, or very similar, results). Per variable, the regression coefficient *B* and its *P*-value were calculated. Note that for multivariate meta-regression analysis, a minimum of 10 studies is required for each covariate [34].

## Results

**Figure 1:**
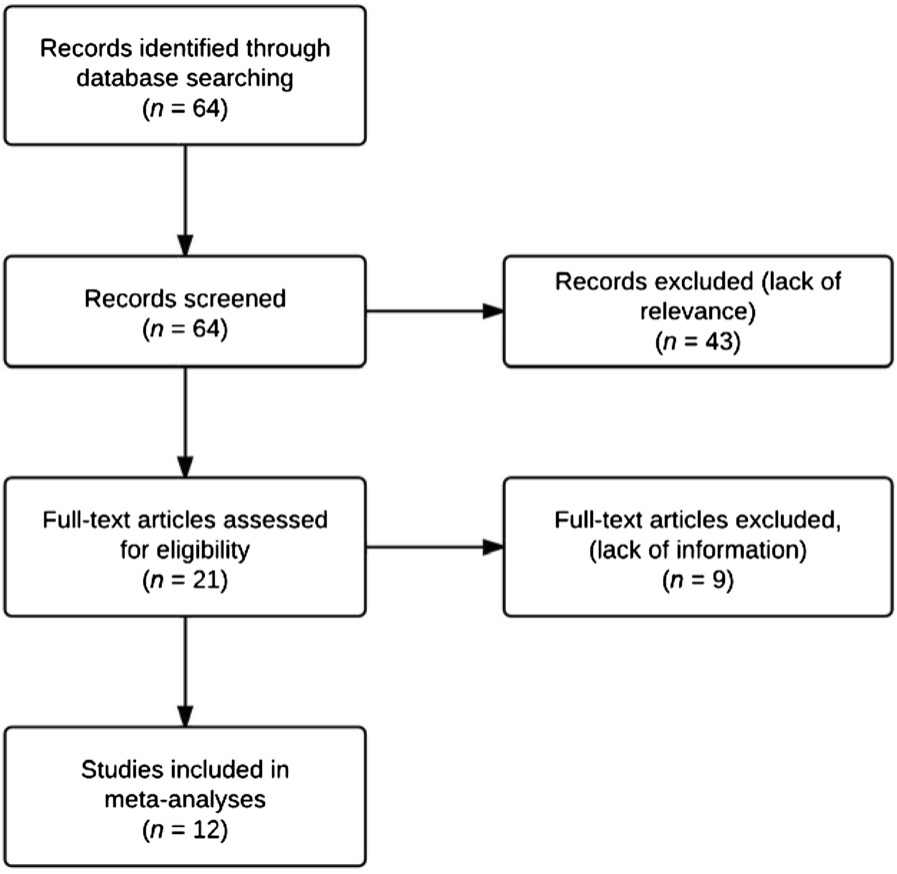
**Study selection **[23]**.**

Initially, the search yielded 64 papers (Figure 1). The reviewers agreed on 20 eligible studies, disagreeing 4 times (*kappa* = 0.64, indicating “good agreement” [35]). Open discussions led to one more eligible paper, whereupon two authors designated 12 papers as “definitely relevant”.

Data on high tibial osteotomy [36,37], or healthy subjects [38], were discarded. When the studies presented different subgroups (e.g., receiving various physical therapy regimens [39,40], or patients with varying severity of osteoarthritis [41]), these were regarded as different pre-post comparisons, which led to 16 *independent* comparisons, labeled as “studies” (cf. Table 1, which includes six references [42-47] not mentioned in the text so far). No untreated control group was used in any of the studies. Of the 16 studies, 9 were concerned with TKA, 6 with UKA, and 1 with a mix (12% UKA). Several studies had more than one measurement after the operation, from 0.5–60 months post-operatively, which added 13 *dependent* comparisons.

**Table 1:** Main characteristics of the studies analyzed; numbers in the first column refer to pre- versus post-arthroplasty comparisons with different subjects, and letters to different post-operative measurement times

	**Study (reference)**	***N***^**a**^	**Arthro-** **plasty**^**b**^	**Time post** **(months)**^**h**^	**Distance walked(m)**	**Walking Speed**		**Gender** **m/f**^**i**^	**Age**^**j**^	**BMI**^**j**^
						**pre**	**post**			
1	Berman, 1987 [41]	B 16 A 16	TKA	18	3.8	0.58 ± 0.2	0.83 ± 0.2	NA	64.80	
2		B 12 A 12	TKA^c^	24	=	0.59 ± 0.2	0.71 ± 0.3	NA		
3a	Kroll, 1989 [42]	B 18 A 18	TKA	5	10	0.84 ± 0.2	0.98 ± 0.2	7/11	68	27.43
3b		B 18A 18	=	13	=	0.84 ± 0.2	1.07 ± 0.1	=		
4	Mattsson, 1990 [43]	B 20A 20	UKA	12	249.6	1.04 ± 0.2	1.24 ± 0.1	11/9	63 ± 4.5	
5	Ivarsson, 1991 [44]	B 10A 10	UKA	6	10	0.83 ± 0.3	0.93 ± 0.2	4/6	64 ± 5	
6	Weidenhielm, 1993 [36]	B 36A 36	UKA	12	5	1.03 ± 0.2	1.19 ± 0.2	18/18	64 ± 5	28.1
7	Weidenhielm, 1993 [39]	B 19A 19	UKA^d^	3	5	1.13 ± 0.1	1.14 ± 0.1	11/8	64 ± 4	30.08
8		B 20A 20	UKA	3	=	1.09 ± 0.2	1.17 ± 0.1	11/9	63 ± 5	29.07
9a	Fusi, 2002 [45]	B 16A 13	TKA	2	255	0.85 ± 0.2	0.81 ± 0.2	13/3 NA	72 ± 3.6	29.6 ± 5.2
9b		B 16A 8	=	6	243	0.85 ± 0.2	1.07 ± 0.2	=		
9c		B 16A 10	=	12	321	0.85 ± 0.2	0.98 ± 0.2	=		
10a	Parent, 2002 [46]	B 65A 65	TKA	2	10	0.80 ± 0.3	0.70 ± 0.2	25/40	68.6 ± 8.7	31.2 ± 5.6
10b		B 65A 64	=	4	=	0.80 ± 0.3	0.90 ± 0.3	25/40 24/40		
11a	Lamb, 2003 [47]	B 79A 68	mixed	3	5	1.10 ± 0.5	1.34 ± 0.4	40/39 36/32	71.1 ± 6.4	29.0 ± 3.9
11b		B 79A 57	=	6	=	1.10 ± 0.5	1.45 ± 0.5	40/39 29/28		29.1 ± 3.8
12a	Börjesson, 2005 [37]	B 22A 22	UKA	3	5	1.07 ± 0.2	1.16 ± 0.2	11/11	63 ± 4	28.0
12b		B 22A 22	=	12	=	1.07 ± 0.2	1.24 ± 0.2	=		
12c		B 22A 22	=	60	=	1.07 ± 0.2	1.19 ± 0.2	=		
13	Mandeville, 2007 [38]	B 21A 21	TKA	6	10	0.89 ± 0.2	1.05 ± 0.2	6/15	62.6 ± 7.3	32.6 ± 5
14a	Rahmann, 2009 [40]	B 20A 17	TKA^e^	0.5	=	0.71 ± 0.4	0.49 ± 0.3	NA 5/12	70.4 ± 9.2	28.8 ± 6.2
14b		B 20A 17	=	3	=	0.71 ± 0.4	1.00 ± 0.4	=		
14c		B 20A 17	=	6	=	0.71 ± 0.4	0.98 ± 0.3	=		
15a		B 24A 17	TKA^f^	0.5	10	0.99 ± 0.2	0.67 ± 0.3	NA	69.4 ± 6.5	28.4 ± 4.6
15b		B 24A 17	=	3	=	0.99 ± 0.2	1.14 ± 0.3	NA		
15c		B 24A 14	=	6	=	0.99 ± 0.2	1.25 ± 0.3	NA		
16a		B 21A 19	TKA^g^	0.5	10	0.76 ± 0.4	0.57 ± 0.2	NA	69 ± 8.9	28.0 ± 4.1
16b		B 21A 19	=	3	=	0.76 ± 0.4	1.03 ± 0.3	NA		
16c		B 21A 17	=	6	=	0.76 ± 0.4	1.09 ± 0.2	NA		

= Same as immediately above.

NA not available in the paper.

^a^* N* number of subjects, B before surgery, A after surgery.

^b^*TKA* total knee arthroplasty, *UKA* unicompartmental knee arthroplasty.

^c^ Patients also having contralateral radiographic signs of knee osteoarthritis.

^d^ Patients undergoing post-operative physiotherapy.

^e^ Standard post-operative care ("ward ").

^f^ Post-operative aquatic treatment.

^g^ Post-operative water exercises.

^h^ Number of months after the operation (post-operative measurement).

^i^*m* male, *f* female.

^j^ Left blank where no information was given.

Where relevant, data are given as mean ± standard-deviation.

### Subject characteristics

The studies involved 419 patients. Information on gender was provided in 12 studies, with 45.7% male subjects. Age was provided in all studies, with a pre-operative mean of 67.2 years. BMI was reported in 12 studies, with a mean value of 29.3. Disease severity, co-morbidity, pain, and function were each reported in less than 10 studies, and were not taken into consideration in further analyses.

### Methodological characteristics

Information on distance walked was provided in all studies. Most studies used a walkway, varying from 3.8–10 m, but two studies specified time walked. Multiplying these times by mean walking speed at the time of measurement, showed distances walked from 243–321 m. Pre-operative self-selected walking speed varied widely, from 0.58 m/s to 1.13 m/s (mean value 0.93 m/s).

None of the papers provided a correlation between pre- and post-operative values. One paper reported [46], the *t*-value of a paired *t*-test, corresponding to an *r*_*P*_ of 0.537 [25], which was used for all initial calculations.

### Meta-analyses

**Figure 2:**
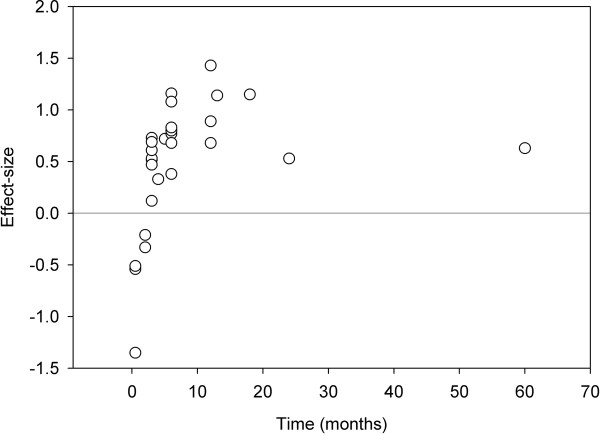
Effect-sizes (vertical axis) against time (horizontal axis) in all comparisons; of the 29 comparisons, 16 were independent.

Effect-sizes were calculated for all comparisons, and depicted against post-operative measurement time (Figure 2). The pattern of the first five months suggested an increase in walking speed, with considerable heterogeneity. The highest effect-size was found at 12 months, after which time some decline became apparent. Therefore, 0.5–5 months after arthroplasty was determined as “short-term”, 6–12 months as “mid-term” and 13 months or more as “long-term”.

**Figure 3:**
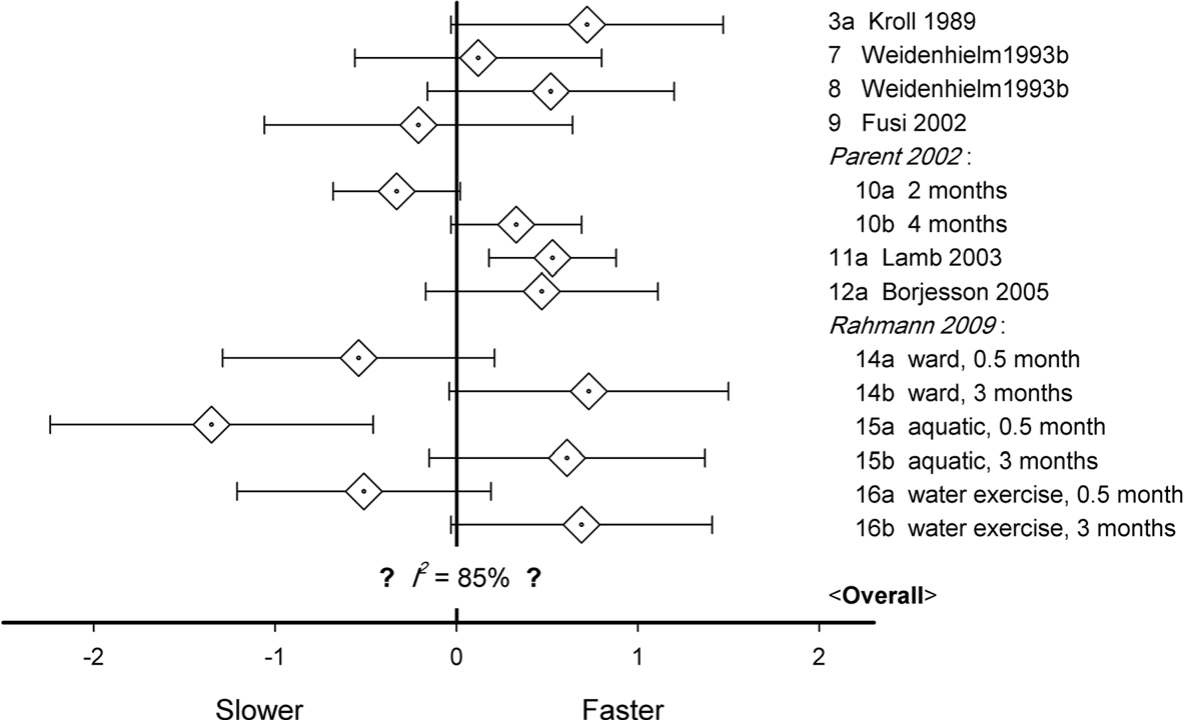
**Walking speed effect-sizes in the first 0.5–5 months after knee arthroplasty with mean values (◊) and 95% confidence intervals (horizontal error bars); given the large between-study variance, no valid overall estimate was possible; note that Weidenheim **[39]** followed two different groups.**

Ten studies reported short-term walking speed. Effects varied from significantly slower to significantly faster (Figure 3). In four studies, two post-operative values were provided. Since meta-analysis requires independent data, each of these four studies could be used in two different meta-analyses, which resulted in 2^4^ = 16 possible meta-analyses. These were all performed. Heterogeneity was always significant (mean *Q* = 59.7, range 52.8–66.7, *P*-values < 0.001), and random models were used. The mean *ES* equaled 0.21 (range −0.03 to 0.45), with *P*-values from 0.87 to < 0.001. In 4 of the analyses, the effect-size was significant (*P* < 0.05). Since between-study variance was large, with mean *I*^2^ 85.1% (84.8–85.7%), no valid estimate of the short-term effect-size could be reached.

**Figure 4:**
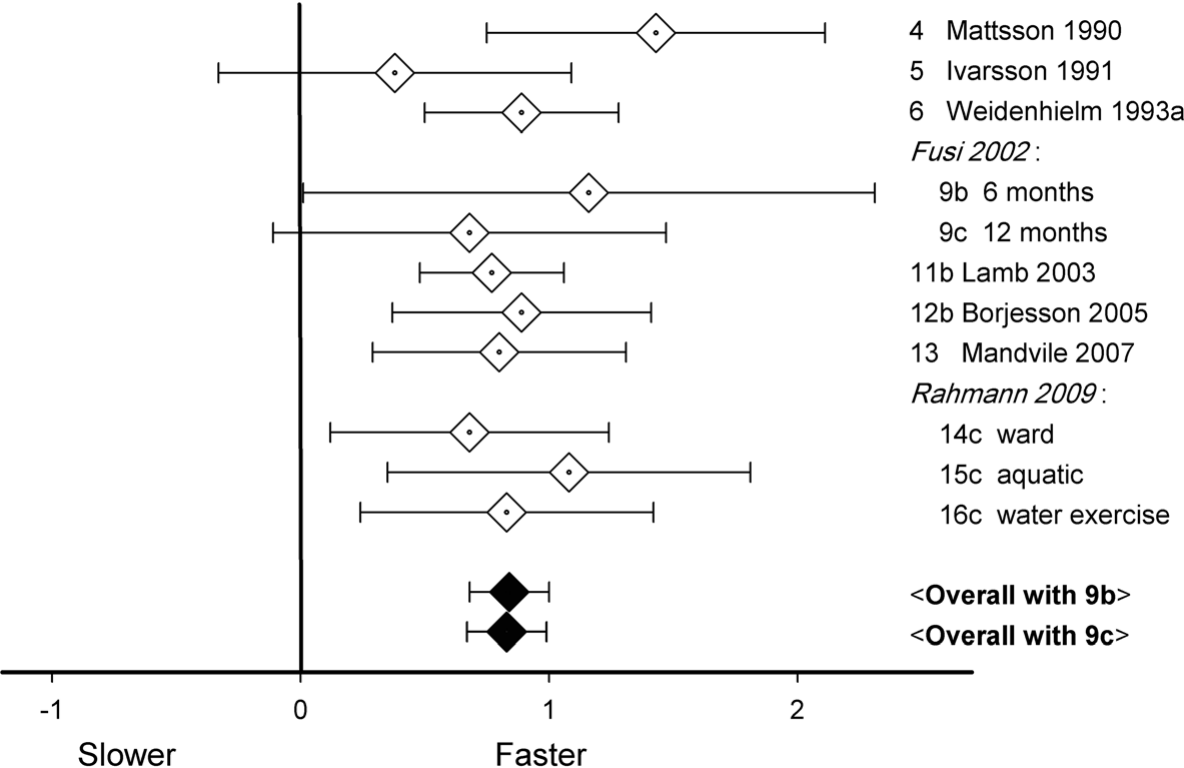
Walking speed effect-sizes 6–12 months after knee arthroplasty; bottom lines: overall effect-sizes, 95% CI 0.68–1.00 (with 9b), or 0.67–0.99 (with 9c).

Ten studies gave mid-term walking speed. Effects varied from non-significantly to significantly faster (Figure 4). In one study, two post-operative values were given (9b and 9c, for study numbers, see Table 1), implying two ways to perform the meta-analysis. The mid-term effect-size equaled 0.84 (with 9b) or 0.83 (with 9c). There was no significant heterogeneity (*Q*-values < 6.0, *P*-values > 0.7), and fixed effect models were used. Both estimates of the middle-term effect-size were significant (*P*-values < 0.001). In the studies used for these analyses, walking speed increased from 0.96 m/s pre-operatively to 1.16 m/s after 6–12 months. Since *I*^2^ equaled 0%, there was no sign of between-study heterogeneity.

**Figure 5:**
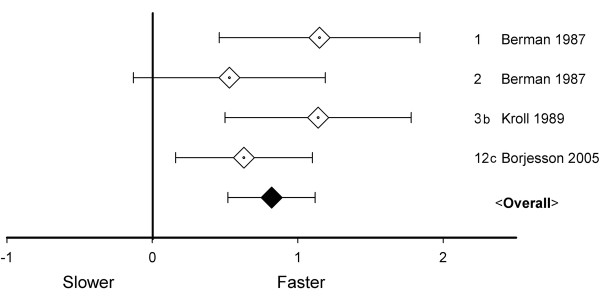
**Walking speed effect-sizes 13–60 months after knee arthroplasty; overall effect-size, 95% CI 0.52–1.12; note that Berman **[41]** followed two different groups.**

The four studies with measurements more than 12 months post-operatively were independent. Effects varied from non-significantly to significantly faster (Figure 5). The long-term effect-size equaled 0.82, there was no significant heterogeneity (*Q* = 3.2, *P* > 0.1), and a fixed model was used, which revealed the effect-size to be statistically significant (*P* < 0.001). In the studies used for this analysis, walking speed increased from 0.80 m/s pre-operatively to 1.02 m/s more than 12 months post-operatively. *I*^2^ equaled 5.5%, implying low heterogeneity [31].

Rerunning the above analyses for *r*_*P*_-values 0.0–0.9 provided largely similar results. For the first five months post-operatively, heterogeneity was sometimes lower (70%–75% for *r*_*P*_ 0.0–0.1), but still mostly “high”. For later measurement times, *ES*s differed by not more than 7% from the initial estimates. The original calculations were used for the conclusion. For 6–12 months post-operatively, heterogeneity was sometimes non-zero (3%–28% for *r*_*P*_ 0.8–0.9), but still “low”. There was, however, one noticeable difference, for 13–60 months post-operatively, with heterogeneity turning from “low” to “moderate” (41%–54%) for *r*_*P*_ 0.8–0.9.

### Meta-regression

In the first five months post-operatively, between-study heterogeneity was high, and we performed univariate meta-regressions of post-operative time (months), age (years), distance walked (m), UKA vs. TKA (1 vs. 0), gender (% male), BMI, and pre-operative walking speed (m/s). We used the first post-operative measurements of the relevant studies. In the initial calculations (Table 2), the only significant factor was time (*B* = 0.39, *P* < 0.001), with larger effect-sizes at later times. Significant positive regression with time was also found in the reruns with correlations 0.0–0.9.

**Table 2:** **Meta-regression of effect-sizes 0.5–5 months post-operatively with number of comparisons used in the analysis (*k*), percentage between-study variance (*I***^**2**^**), the regression coefficient (*B*), and its*P*-value (*P*); from papers with more than 1 short-term comparison, only the first one was selected**

	***k***	***I***^***2***^	***B***	***P***
Time	10	85.3	0.39	< 0.001
Age	10	85.3	−0.08	0.14
Distance walked	10	85.3	−0.001	1.0
UKA	10	85.3	0.63	0.12
Gender (% male)	10	85.3	−0.001	0.99
BMI	10	85.3	−0.05	1.0
Initial speed	10	85.3	1.82	0.14

For 6–12 months post-operatively, heterogeneity remained low upon recalculation. Therefore, we did not calculate a meta-regression for this time period.

For measurements 13–60 months post-operatively, heterogeneity was low or moderate. This may warrant meta-regression, but [34] there were only four studies. Still, the question remains if functional recovery lasts over time, or starts to decline later on. Meta-regression of time and time squared was performed for all last measurement points in the complete post-operative period. In the initial calculation, there was a positive effect of time (*B* = 0.06, *P* = 0.02) and a negative effect of time squared (*B* = −0.001, *P* = 0.02), suggesting an initial increase of the effect-size, but a small decrease later on. In all reruns, this pattern remained similar.

## Discussion

We performed meta-analyses of 16 studies of self-selected walking speed in 419 patients with knee osteoarthritis before versus 0.5–5 (short-term), 6–12 (mid-term), and 13–60 (long-term) months after unicompartmental or total knee arthroplasty. None of the studies used an untreated control group.

In the short term, between-study heterogeneity was too large for a valid estimate of the overall effect-size. Mid-term heterogeneity was absent or low, and long-term was either low or moderate. In both latter measurement periods, patients walked 0.8 standard-deviations faster than pre-operatively (*P* < 0.001).

In meta-regression of the short-term data, later measurement time coincided with more effect (*P* < 0.001). Moreover, meta-regression of the last measurement points of all studies revealed a positive effect of time, and a negative effect of time squared (both, *P* = 0.02), suggesting an initial increase over time, and a decrease later on.

### Causality

The lack of randomized control implies that there was “no evidence from trials” [48]. Indeed, RCTs are relatively rare in surgery [49]. Still, RCTs are certainly desirable to improve our understanding of the walking speed effects of arthroplasty. For the time being, we may conclude that patients walk faster after knee arthroplasty, while any causal inference would be beyond the present study.

### Large improvement

Mid-term and long-term effect-sizes were in the order of 0.8 standard-deviations. This is a “large” effect [50], and the question is: Do post-arthroplasty patients return to the level of their healthy peers? Walking speed in knee osteoarthritis was reported to be 0.16 m/s below that of healthy peers [9]. In the present meta-analysis, it improved by 0.20–0.22 m/s, which does suggest that walking speed may normalize after knee arthroplasty. A difference of 0.1 m/s has been proposed as the minimum “meaningful” difference [10], and the average improvement found was clearly larger. Still, reliable meta-analytic information on pre-operative walking speed in osteoarthritis patients vs. their healthy peers will be necessary to further evaluate the above suggestion of “normalization”.

### Rapid functional recovery

Considerable short-term heterogeneity of effect-sizes was found. At later times, post-operative improvement reached a plateau, which is in agreement with the literature e.g., [27], such as the observation of Gandhi et al. that outcomes of total knee replacement are “relatively constant for 3–4 years after surgery” [28], p. 15. Clearly, different patients recover from the operation, or learn to walk with an artificial joint, at varying speeds before they reach a plateau. Hence, “rapid recovery” [51,52] is a relevant topic for research.

Time was found to be a significant predictor of short-term functional recovery, but many potential predictors of post-operative walking speed were not mentioned often enough in the studies analyzed. There was a positive regression of UKA on initial recovery, but it did not reach significance. Post-operative physical therapy may be beneficial 3–4 months post-operatively [53], but in the present meta-analysis, sufficiently precise information on post-operative regimens was not given in enough studies. In the literature, co-morbidity was reported to predict walking speed 2 months after the operation [54], but this correlation was no longer present when pre-operative walking speed was included in the model. In the present study, pre-operative co-morbidity was not reported sufficiently often, but initial speed had a positive regression on post-operative speed, again, however, without significance.

### Lasting functional recovery?

As predicted, meta-regression over the last measurement points of all studies suggested a long-term decline of walking speed. Since the last measurement point may have had a large influence on this result (Figure 2), we recalculated the effects of time without this measurement after 60 months, and still found a positive effect of time (*B* = 0.17, *P* = 0.002), and a negative effect of time squared (*B* = −0.006, *P* = 0.007). Hence, the present paper confirmed the existence of functional decline after a mid-term plateau [28]. On the other hand, the number of long term measurement points was low. Another published study found that early functional advantages of UKA were retained 15 years post-operatively [29]. Therefore, it still remains unclear which factors may enhance lasting functional recovery.

### Clinical relevance

The present study is, to the best of our knowledge, the first meta-analysis of the effects of knee arthroplasty on walking speed.

Patients tend to have overoptimistic expectations of functional recovery after arthroplasty, and to underestimate recovery time [55]. Moreover, it is not clear when exactly surgeons decide to recommend joint replacement [56], and provider-patient agreement on the expected benefits and risks of knee replacement is often low [57]. The present meta-analysis reveals that, on average, walking speed will increase considerably after knee arthroplasty, but this may take several months to occur. Moreover, in the long term, walking speed may decline again, which could be a sign of increasing co-morbidity [3].

The physiology of comfortable walking speed remains largely unknown. Just as in breathing rate, it is easy to change walking speed at will, but, again like breathing rate, it is prone to fall back to its own intrinsic parameters. Walking speed is a fair predictor of, e.g., co-morbidity, atherosclerosis, inflammatory status, cognitive impairment, hospitalization, and even mortality [4], and matches the predictive value of extensive clinical evaluation [4]. Walking speed is certainly not specific, but it is easy to measure, provided this is always done with the exact same methodology [14], including, for instance, the amount of clutter in the walkway [58].

Traditionally, UKA was used for older, inactive patients with medial knee osteoarthritis [59]. To date, however, the boundaries have become blurred, and there is considerable overlap in indications for UKA or TKA [60]. In the present meta-regression analysis, UKA led to somewhat better results than TKA, but not significantly so. This is in agreement with the literature [59].

### Limitations

Four databases were used (MEDLINE, Web of Science, the Cochrane Library, and PEDro) with a limited number of search terms. We may have missed relevant papers, authors who found no effects on walking speed may have refrained from reporting it, and other forms of publication bias cannot be excluded. Note, moreover, that the two authors who read the papers in detail did not do so independently. On the other hand, this was a relatively modest study, with straightforward results, that were in agreement with the clinical literature.

There is still some debate in the literature on the appropriateness of using the standard *Q*-statistic in the choice for a random or a fixed effects model [61]. In the present study, visual inspection of the graph, the standard *Q*, and *I*^2^, all led to the same classification of heterogeneous vs. non-heterogeneous subsets of effect-sizes, suggesting that the specific calculation that was used did not affect main results.

An important limitation of the present study is the lack of randomized controlled trials, which precluded causal interpretation. Correlations between pre- and post-test were never explicitly provided, but recalculations suggested that this omission did not affect the pattern of results. The number of studies analyzed was relatively low, and potentially relevant factors were often not mentioned, which made it impossible to reach any firm conclusions as to which factors enhance rapid or lasting functional recovery.

## Conclusion

A meta-analysis of pre and post-arthroplasty walking speed revealed a large effect 6–60 months post-operatively. For the first 0.5–5 months post-operatively, heterogeneity of effect-sizes precluded a valid estimate of short-term effects. Hence, patients may expect a considerable improvement of their walking speed, which, however, may take several months to occur. Moreover, the analysis suggested a small decline from 13 months post-operatively onwards. Such a decline may be a sign of increasing co-morbidity.

## Competing interests

The authors declare that they have no competing interests.

## Authors’ contributions

All authors made substantial contributions to conception and design of the study. HAB, HRFY, and OGM were involved in data acquisition, HAB, HRFY, OGM, HCWdV, SMB, DLK, and JHvD were involved in data analysis and interpretation of data. All authors were involved in drafting the manuscript, and revising it critically. All authors have given final approval of the version to be published.

## Pre-publication history

The pre-publication history for this paper can be accessed here:

http://www.biomedcentral.com/1471-2474/13/66/prepub
